# Diabetic Ketoacidosis Unmasking a Diagnosis of Glucose-6-Phosphate Dehydrogenase Deficiency: A Case Report and Literature Review

**DOI:** 10.7759/cureus.23842

**Published:** 2022-04-05

**Authors:** Umair Ansari, Puja Bhardwaj, Hamza Quadri, Martin Barnes, Jerry George

**Affiliations:** 1 Internal Medicine, Donald and Barbara Zucker School of Medicine at Hofstra/Northwell, Port Jefferson, USA; 2 Hematology and Oncology, Donald and Barbara Zucker School of Medicine at Hofstra/Northwell, Port Jefferson, USA; 3 Hematology and Oncology, New York Cancer and Blood Specialists, Port Jefferson, USA

**Keywords:** non-immune hemolytic anemia, glucose-6-phosphate-dehydrogenase deficiency (g6pd), general internal medicine, clinical hematology, endocrinology and diabetes, diabetes

## Abstract

Glucose-6-phosphate dehydrogenase deficiency (G6PD) is the most common enzyme deficiency. Mode of inheritance is X-linked recessive with a high prevalence in endogamous marriages, such as Jehovah’s Witness. Oxidative triggers such as infection, ingestion of certain medications, certain types of food, and in rare instances diabetic ketoacidosis (DKA) may unmask the diagnosis by triggering a hemolytic event. We describe the case of a 43-year-old male with type 2 diabetes who presented with DKA and subsequently became anemic four days after his admission, with the hemoglobin continuing to fall. After extensive workup, it was found that the patient had G6PD confirmed by a low glucose-6-phosphate dehydrogenase assay. We hypothesized that the oxidative stress from the DKA unmasked G6PD induced hemolysis in our patient. During our literature search, we also noticed that hemolysis was delayed on average by four to seven days in these patients after the initiation of insulin therapy similar to our patient. It is postulated that the delayed onset of hemolysis may be due to high levels of glucose in the blood. Hyperglycemia may offset the effects of G6PD deficiency by increasing the production of G6PD. When the levels of glucose start falling, hemolysis becomes apparent.

## Introduction

Glucose-6-phosphate dehydrogenase deficiency is the most common enzyme deficiency in humans [1]. We can trace the epidemiological origins of the disease by looking at the many electrophoretic variants. The G6PD B variant is present worldwide and has normal activity. The two other most widely documented mutated variants are G6PD A, seen in sub-Saharan Africa, and G6PD med seen in the Mediterranean region [2]. Hence the reported cases are also the highest in these regions or in those whose ancestors originated from here [3].

The mode of inheritance for this disease is X-linked recessive, essentially rendering all communities with a high prevalence of endogamous marriage, such as Jehovah’s Witness, at an increased risk. Severe hemolysis in this population is particularly cumbersome to manage since they decline therapeutic use of blood and blood products for religious reasons [4].

A patient with G6PD deficiency may remain asymptomatic for a long period of their life, hence undiagnosed for much of their youth. Oxidative triggers such as infection, the ingestion of certain medications like antimalarials, certain types of food, and in rare instances diabetic ketoacidosis may unmask the diagnosis by triggering a hemolytic event [5]. Although only a handful of patients have been reported to have G6PD induced hemolytic anemia in the setting of DKA, such as our patient, physicians must be aware of the possibility and be prepared to manage accordingly.

## Case presentation

A 43-year-old African American male with type 2 diabetes, who was a Jehova’s witness, presented to the emergency department with complaints of vomiting, fever, increased thirst, and urinary frequency for one day. On further questioning, he was non-compliant with his medications and had an ill contact at a care home, where he worked as a nursing assistant. His vitals and presenting labs are shown in Table 1. The patient was afebrile, tachypneic, tachycardiac, and hypertensive on presentation. The hemoglobin and hematocrit were both elevated. The patient also had leukocytosis. Fasting blood glucose was also elevated on presentation and urine ketones were present. Lipase was normal. The electrocardiogram (EKG) did not show any ST-T changes and troponins were normal. The past family history was significant for sickle cell trait. The patient also had a primary metabolic acidosis with a secondary respiratory alkalosis. The patient was subsequently admitted to the ICU for diabetic ketoacidosis and treated with a protocol that included regular insulin infusion.

**Table 1:** Presenting vital signs, labs, and arterial blood gas.

Vital signs		
Parameter	Value	Reference range
Temperature	97.6°F	97-99°F
Blood pressure	133/88 mmHg	<120/80 mmHg
Heart rate	123 bpm	60-100 bpm
Respiratory rate	21 breaths/min	12-16 breaths/min
Oxygen saturation (SpO2)	99% on room air	>92%
Labs upon admission		
Hemoglobin (Hb)	19 mg/dL	13.2-16.6 mg/dL
Hematocrit (HCT)	55.3%	38.3-48.6%
White blood cells (WBC)	19.7 K/uL	4.5-11 K/uL
Fasting blood glucose	426 mg/dL	70-100 mg/dL
Sodium	129 mmol/L	136-144 mmol/L
Potassium	3.9 mmol/L	3.6-5.1 mmol/L
Chloride	94.0 mmol/L	97-110 mmol/L
Bicarbonate	25.5 mmol/L	22-32 mmol/L
Blood urea nitrogen	11.0 mg/dL	8-20 mg/dL
Creatinine	1.1 mg/dL	0.7-1.2 mg/dL
Calcium	9.2 mg/dL	8.9-10.3 mg/dL
Albumin	5.0 g/dL	3.5-5.2 g/dL
Anion gap	43	8-12
Urine ketones	≥80	Negative
Lipase	27 U/L	13-60 U/L
Troponin 0 hours	32 ng/L	=22 ng/L
Troponin 1 hour	26 ng/L	=22 ng/L
Arterial blood gas (ABG)		
pH	7.163	7.38-7.46
pCO2	9.0	32-46
HCO3	3.1	21-29

**Figure 1:**
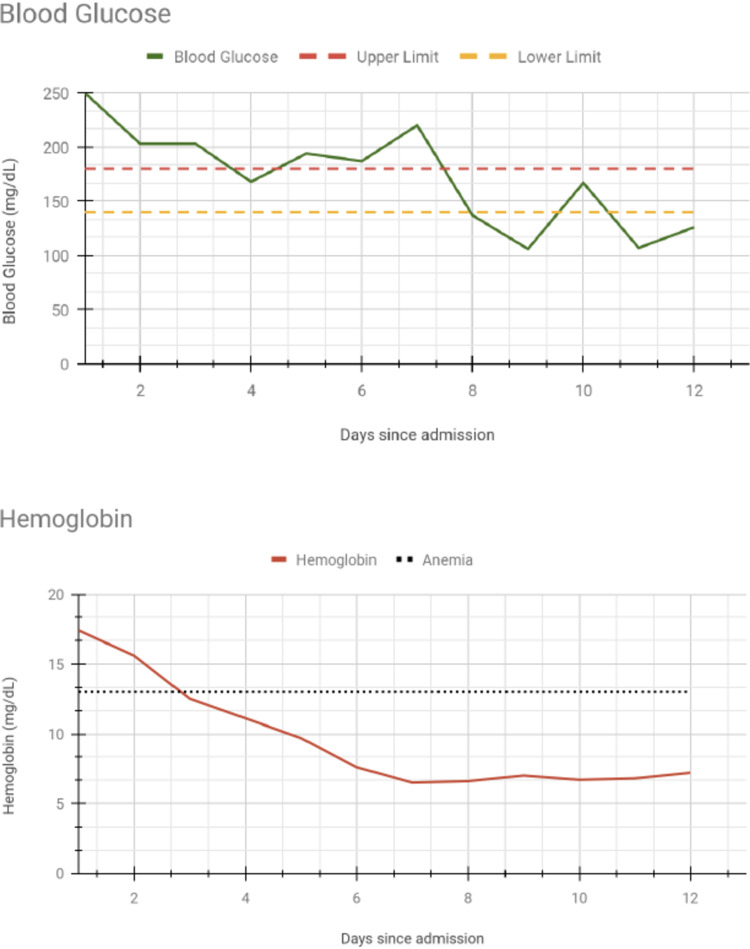
The relationship between blood glucose levels and hemoglobin in our patient.

The patient remained afebrile throughout the hospital stay and the leukocytosis resolved with fluids. On day five of treatment, the anion gap closed and the patient was transitioned from an insulin drip to Lantus. The hemoglobin dipped below the normal range on the fourth day of admission and continued to trend down (Figure 1). The patient started developing lightheadedness and blurred vision eight days into the hospital stay.

This is when hematology was consulted and a hemolytic anemia workup showed an increased LDH, a decreased haptoglobin, an increased corrected reticulocyte count, and a negative Coombs test, indicative of non-immune hemolysis (Table 2). Further testing revealed folate, vitamin B12, thyroid-stimulating hormone (TSH), transferrin saturation, and ferritin levels within normal limits, essentially eliminating megaloblastic anemia, thyroid disease, and iron deficiency anemia as potential causes. Hemoglobin electrophoresis yielded normal adult hemoglobin and osmotic fragility revealed rare spherocytes. A babesia workup was negative as well. Lead levels were also within normal range, which was done because basophilic stippling was seen on the peripheral smear. Flow cytometry revealed normal expression of GPI-linked antigens, ruling out paroxysmal nocturnal hemoglobinuria. Two fecal occult blood tests came back positive on the seventh and eighth days of admissions, we followed this up with an upper endoscopy which revealed mild gastritis and several small gastric polyps, for which he underwent polypectomy. A low glucose-6-phosphate dehydrogenase assay in blood on the eighth day of admission finally shed light on the cause of our patient’s hemolysis. The anemia was eventually treated with epoetin alfa, folate, vitamin B12, and ferritin, because of his refusal to receive a blood transfusion.

**Table 2:** Hemolytic anemia workup.

Test result	Value	Reference range
Lactate dehydrogenase (LDH)	597 U/L	140-280 U/L
Haptoglobin	<10.0 mg/dL	45-165 mg/dL
Reticulocyte count	3.63%	0.5-1.5%
Coombs test	Negative	Negative
Folate	9.37 ng/mL	2.7-17.0 ng/mL
Vitamin B12	1256 pg/mL	190-950 pg/mL
Thyroid-stimulating hormone (TSH)	1.350 mIU/L	0.5-5.0 mIU/L

## Discussion

The first-ever published association between G6PD and hyperglycemia was by Chanmugam and Frumin back in 1964 [6]. Since then, this association has become more established with publications such as Heymann et al., which found an odds ratio of 1.44 (95% CI: 1.145-1.815) (p=0.002) or a study by Niazi, which showed a 12.4% prevalence of G6PD deficiency as compared to the healthy population of 2% (p<0.008) [7,8]. This correlation could be attributed to genes that affect both insulin secretion and G6PD antioxidant properties contributing to a predisposition to diabetes [9].

Although there are only a handful of publications to reflect upon, we did notice that patients would almost never have hemolysis on presentation, but would start to develop it after one or more days into admission like our patient. In a study on twin patients by Haroun, signs of hemolysis like jaundice and pallor were first seen three days after euglycemia was achieved [10]. A study of two female patients with hemolysis by Errico et al. also showed similar findings on days one and four after treatment was started [11]. In a case report by Galtrey and Pathansali, clinical signs of hemolysis didn’t appear until day four of admission in a Kenyan man with hyperglycemic diabetic crisis [12]. A literature review of cases with clinical presentation of hemolysis is demonstrated in Table 3.

**Table 3:** Literature review of the cases reported for DKA-induced G6PD hemolysis. DKA: diabetic ketoacidosis; G6PD: glucose-6-phosphate-dehydrogenase deficiency; M: male; F: female

Reference	Age	Gender	Clinical presentation of hemolysis (days after admission)
Mehta et al. [13]	18	M	Day 3 - reddish discoloration of urine
Galtrey and Pathansali [12]	54	M	Day 4 - hypoxemia, discolored urine, and muscle weakness
Gu et al. [14]	59	M	Day 4 - jaundice and pallor
Azaki and Alalawi [5]	17	M	On admission
Twin 1 - Haroun [10]	4	M	Day 7 - jaundice and pallor
Twin 2 - Haroun [10]	4	M	Day 7 - jaundice and pallor
Agarwal et al. [15]	40	M	On admission
Errico et al. [11]	9	F	Day 4 - pallor, jaundice, and asthenia
Errico et al. [11]	12	F	Day 1 - jaundice

A study by Errico et al. postulated that the reason why hemolysis becomes apparent after the hyperglycemia is corrected is that high levels of glucose in the blood offset the effects of the glucose-6-phosphate dehydrogenase deficiency, by increasing the production of glucose-6-phosphate [11]. This provides a reasonable explanation for why we saw our patient's hemoglobin only dip after the blood glucose levels started normalizing, which is reflected in similar case reports.

A larger study conducted in 1985 disproved the link between diabetic ketoacidosis and the Mediterranean variant of the glucose-6-phosphate dehydrogenase deficiency by citing there to be a lack of unequivocal evidence of a link between the two [16]. This study looked at 15 patients over a span of 12 years and their episodes of diabetic ketoacidosis complicated by hemolysis, inadvertently all cases had a concurrent bacterial infection or ingestion of a hemolytic drug or both, hence confounding the findings. Our patient was African American and most likely had the G6PD A variant of the gene.

During our case report, we were aware of these confounding factors and how they could affect the correlation between our two variables. Even though our patient had leukocytosis at the time of presentation to the ED and had a sick contact, the patient was afebrile and had signs of severe dehydration like a very dry oral mucosa on a physical examination. The patient’s hemoglobin was also elevated and so was the hematocrit. Although bacterial infection cannot be ruled out, a volume-depleted state is a more plausible explanation for these findings. The patient also had a family history of sickle cell trait, but the hemoglobin electrophoresis came back as normal. Gastrointestinal bleed was also found in the patient due to the presence of many polyps but does not explain the hemolytic and acute pattern of the patient’s findings. Also of note, the patient’s ingestion of a known hemolytic drug either before the presentation or during hospitalization was not found.

## Conclusions

We have yet to affirmatively prove the effects of diabetic ketoacidosis on the unmasking of G6PD hemolysis in a large-scale study, but we have come a long way since the association was first described. Perhaps, if more clinicians were aware of this possibility, especially with certain cohorts of patients, e.g., the Sardinians and African Americans, where there is a high percentage of G6PD deficiency, then insulin therapy and G6PD screening could be utilized more appropriately.
